# Primary hepatic neuroendocrine neoplasm: Long-time surgical outcome and prognosis

**DOI:** 10.1097/MD.0000000000011764

**Published:** 2018-08-03

**Authors:** Changying Shi, Qian Zhao, Binghua Dai, Feng Xie, Jiamei Yang

**Affiliations:** a Department of Hepatobiliary Surgery; b Department of Pathology, Eastern Hepatobiliary Surgery Hospital, Shanghai, China.

**Keywords:** liver, neuroendocrine neoplasm, primary

## Abstract

Primary hepatic neuroendocrine neoplasms (PHNENs) represent a kind of rare liver tumor and its clinical features and prognosis remain unclear. This study aims to reveal the long-term therapeutic outcome of PHNEN and to present its prognostic feature.

A retrospective designed, single-center study containing 22 patients with PHNENs receiving surgical resections was done. Clinical data were reviewed and long-term follow-up was updated. Survival analysis was tried to find the prognostic factors.

Nine patients recurred (recurrence rate = 40.9%) and 6 patients died on the disease. The actual 1-, 3-, and 5-year recurrence-free survival rate were 86.4%, 63.6%, and 52.9%, respectively. The 1-, 3-, and 5-year overall survival rate were 95.5%, 81.8%, and 64.7%, respectively. Median overall survival for group G1, G2, and G3 were 69, 67, and 42 months, respectively.

Patients with PHNEN can have a long survival after radical surgical resection, especially when the tumor proliferative grade exhibits lower (G1/2).

## 1 Introduction

Neuroendocrine neoplasms (NENs) are primary malignant tumors that arise from neuroendocrine cells, which grow throughout the body; as a result, NENs can develop in many different locations.^[1]^ The most common primary organ for these tumors is the gastrointestinal tract, followed by the respiratory system and the thymus.^[2]^ Primary hepatic neuroendocrine neoplasms (PHNENs) are extremely rare, with less than 200 cases reported throughout the English-language literature since Edmondson first described this disease in 1958.^[3–17]^ It has been suggested that PHNEN cells originate from ectopic pancreatic and/or adrenal tissue in the liver or from scattered neuroendocrine cells in the intrahepatic biliary epithelium.^[15,18]^ Another hypothesis is that the neuroendocrine differentiation of a single malignant stem cell provides the major contribution to the genesis and morphology of PHNENs.^[19]^ Prior reports have primarily described several detailed characteristics of this disease, and information is less available regarding surgical outcomes, while hepatic resection has been established as the mainstay treatment for liver neoplasms. Park et al^[17]^ reported on a series of 12 PHNEN patients for whom the median overall survival (OS) was 16.5 months; however, only 3 of these patients underwent surgery. Wang et al^[20]^ analyzed 10 PHNEN cases and reported a 6-year long-term survival rate of 33.6%. No more data can be learned especially regarding surgical outcome, prognostic factors, recurrence pattern, and subsequent treatment. The World Health Organization (WHO) revised its pathological grading system for NENs in 2010, while PHNENs were not mentioned separately. Thus further validation is needed to determine whether cellular proliferation index valued by Ki67 calculation and mitotic grade can be applied in malignancy assessment and survival prediction in PHNEN. We reviewed the total 22 cases of PHNEN who got surgical treatment in author's institute to further study the clinical features, surgical outcome, potential prognostic factors, and subsequent treatment after recurrence of the disease. We hope this study will help us learn more about this rare disease and promote the therapeutic outcome.

## 2 Methods

### 2.1 Patients

From January 2007 to December 2013, a total of 179 patients underwent surgical resection for their liver neoplasms, which were pathologically diagnosed as NENs in Eastern Hepatobiliary Surgery Hospital in Shanghai. Among these, 22 patients were clinically diagnosed as PHNEN after excluding the possibility of metastatic tumors. Their in-hospital records and follow-up data were reviewed. Paraffin embedded tissues were prepared for 4-μm serial sections to re-examine. This study was approved by the medical ethics committee of Shanghai Eastern Hepatobiliary Surgery Hospital.

### 2.2 Follow-up and statistical analysis

A postoperative follow-up was updated to May 2017 via outpatient services or phone interviews. Information regarding recurrence-free survival (RFS), therapeutic modalities after recurrence, and OS was collected (calculated by month). To assess the impact of cellular proliferation, we set low proliferation group for G1/G2, which was compared with high proliferation group (G3) on survival. Potential influencing factors, including age, gender, manifestation, liver disease background, tumor characteristics, radical surgery, and pathological parameters, were collected and assessed. Risk factors of OS and RFS were explored. Cumulative survival comparison between groups was performed with log-rank. Multivariate analysis for independent prognostic factors was determined by Cox proportional hazards model. The Statistical Package for the Social Sciences (version 19.0; SPSS, Chicago, IL) was utilized for statistical analyses.

## 3 Results

### 3.1 Patients’ clinical characteristics

The median age was 49 years (range: 37–82 years), with 12 male and 10 female. Six patients had a history of hepatitis B infection. No history of digestive tumors was recorded. Clinical manifestation included abdominal pain in 6 patients, weight loss in 1, and fatigue in 1. The other 14 patients exhibited no symptoms. No patient reported signs of carcinoid syndrome or other special symptoms.

### 3.2 Laboratory and imaging findings

Liver function were A class of Child–Pugh score and serum AFP and CEA were negative for all. Serum CA19-9 levels were slightly elevated in 2 patients (58.0 and 47.8 IU/L) and turned normal postoperatively. Two patients had a history of benign colonic polyps, which got endoscopic resection and pathological confirmation. In preoperative evaluation, ultrasonography displayed high echogenicity in 18 patients, mixed echogenicity in 3, and low echogenicity in 1, respectively. All patients were assessed by dynamic computed tomography (CT; 21 patients) or magnetic resonance imaging (MRI; 8 patients) scanning. The most typical tumor imaging displayed cystic area with rim enhancement in arterial phase (appeared in 17 patients). Early enhancement of the solid area followed by rapid wash-out appeared in 13 patients. 18F-FDG PET/CT was used to detect latent primary tumor or to exclude extrahepatic lesion in 3 patients in whom fluorodeoxyglucose uptake was positive in hepatic neoplasm. No evidence hint that extrahepatic tumor existed in this group.

### 3.3 Diagnoses

No patients were correctly diagnosed with PHNENs before postoperative pathological examinations. Hepatocellular carcinoma (HCC; 8/22), intrahepatic cholangiocarcinoma (iCCA; 5/22), and cystic tumor with malignant potential (3/22) were mainstay of preoperative misdiagnosis due to a similarity in imaging findings to those tumors. To be noted, most preoperative diagnoses were made doubtfully due to the atypical clinical features. Despite all this, surgical indications were supported by the malignant patterns of tumor growth. In addition to the pathological finding, the final diagnosis would not be made until exclusion of metastatic neuroendocrine tumors from other organs after a period of follow-up.

### 3.4 Tumor characteristics and surgical procedures

Thirteen patients presented multiple lesions and the rest presented solitary ones. Surgery plans were made upon tumor location and burden. Ultimately, 15 patients received radical resection, whereas the remaining 7 patients took debulking resections. No operative deaths occurred. The average length of hospitalization was 9.8 ± 2.8 days (range: 7–13 days). Detailed information regarding patients’ clinical characteristics, surgical procedures, pathological information, and follow-up data is summarized in Table 1.

**Table 1:**
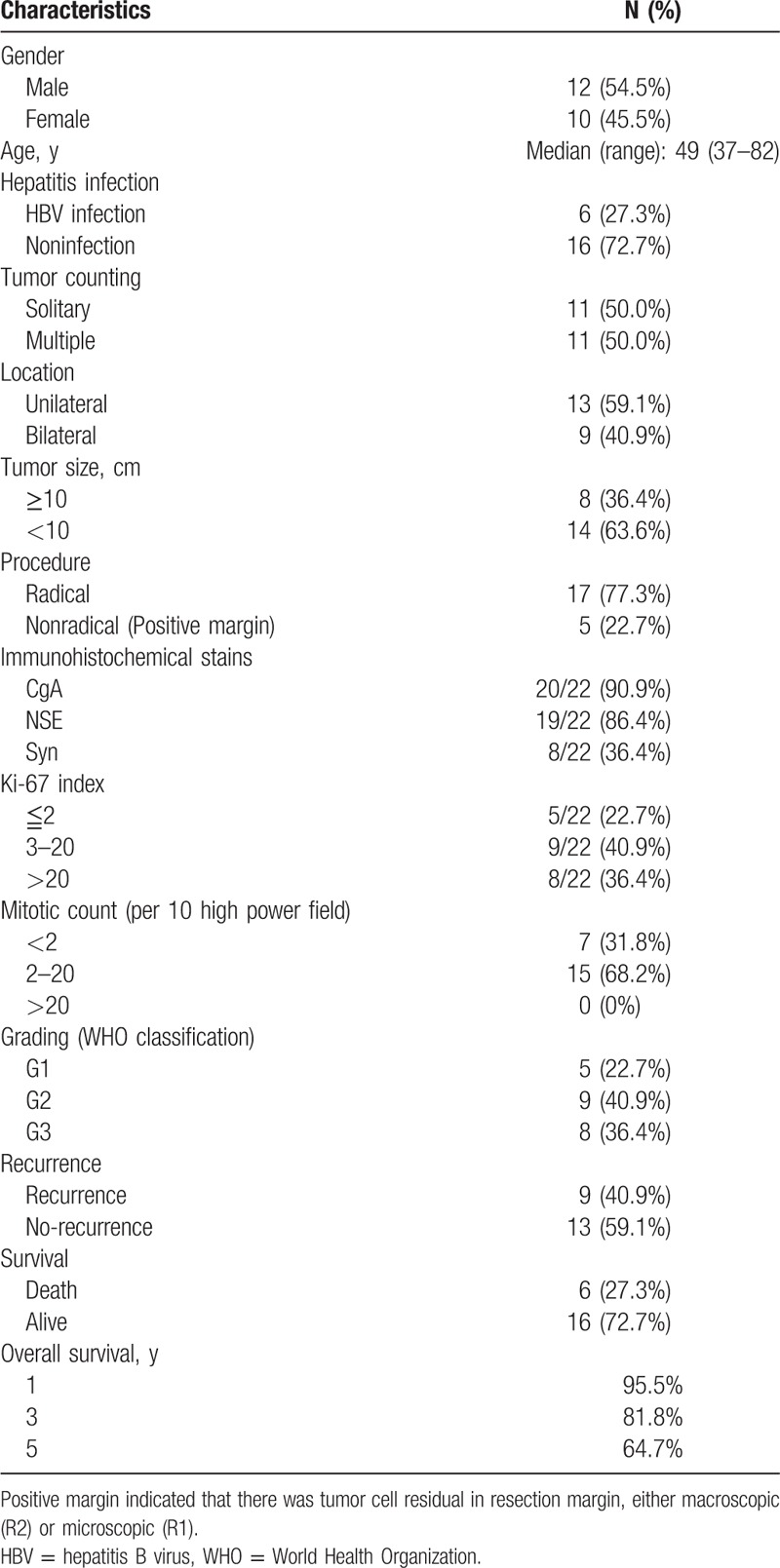
Characteristics of 22 cases of PHNEN.

### 3.5 Pathology and proliferative grade

The size of patients’ nodules ranged from 3.2 to 17.0 cm in diameter. The proliferative grade was classified as G1 in 5 patients, G2 in 9 patients, and G3 in 8 patients, respectively. The mitotic and Ki67 grades were concordant for all 5 G1 patients but discordant for 2 of the 9 G2 and all of the 8 G3. In these discordant patients, the mitotic grade was lower than the Ki67 grade for all.

### 3.6 Follow-up and treatment after recurrence

The minimal length of follow-up was 41 months with a 100% follow-up rate. Nine patients recurred (recurrence rate = 40.9%) and 6 patients died on the disease. The actual 1-, 3-, and 5-year recurrence free survival rate were 86.4%, 63.6%, and 52.9%, respectively. The 1-, 3-, and 5-year OS rate were 95.5%, 81.8%, and 64.7%, respectively. Among the recurrence, 4 patients developed fatal recurrence with a rapid growth of tumor and intrahepatic spreading. The median OS for this subgroup was 14 months. The other 5 patients with local recurrence received comprehensive treatment, including ablation, systemic chemotherapy, and radiotherapy. They got a median OS of 59 months.

### 3.7 Survival analysis and risk factors

Median OS for group G1, G2, and G3 were 69, 67, and 42 months, respectively. Analyses revealed that gender, age, hepatitis, tumor amount, and location have a small-scale relationship with recurrence or survival. Radical surgery improved RFS (*P* = .004) but had no influence on OS (*P* = .072). Tumor size was a risk factor for OS (*P* = .03) but not for RFS (*P* = .055). G3 significantly related RFS (*P* = .007) and nearly related OS (*P* = .050). Multivariate analyses only demonstrated that G3 was an independent factor for RFS (*P* = .019).

## 4 Discussion

PHNEN is extremely rare and unfamiliar to most physicians. PHNEN appears mostly in the 4th and 5th decades, although it may occur in every period of life (from 37 to 82 in our data). No broad gender gap is observed. With respect to hormonal activity, NENs are categorized as functional NENs or nonfunctional NENs. It was reported that PHNENs were associated with a low incidence of symptoms related to hormonal secretion.^[21]^ In our data, no patient was admitted with complaints of carcinoid syndrome, such as flushing, diarrhea, low arterial pressure, Zollinger–Ellison syndrome, or neuropathic hypoglycemia, consistent with former report.^[17]^ Insufficient production of hormones such as serotonin and its metabolic products or a functional deficit in PHNENs may be responsible for the low incidence of hormonal secretion-related manifestations.

**Figure 1:**
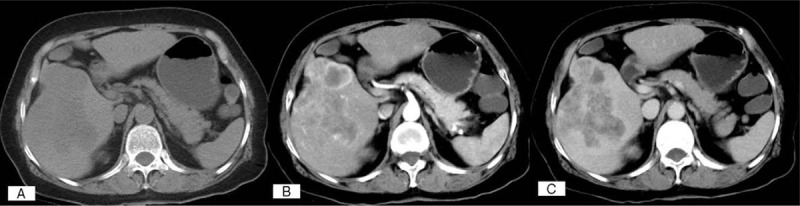
Dynamic CT scans of a PHNEN (G2), which exhibits early arterial enhancement followed by wash-out in the venous phase, similar to a typical HCC.

**Figure 2:**
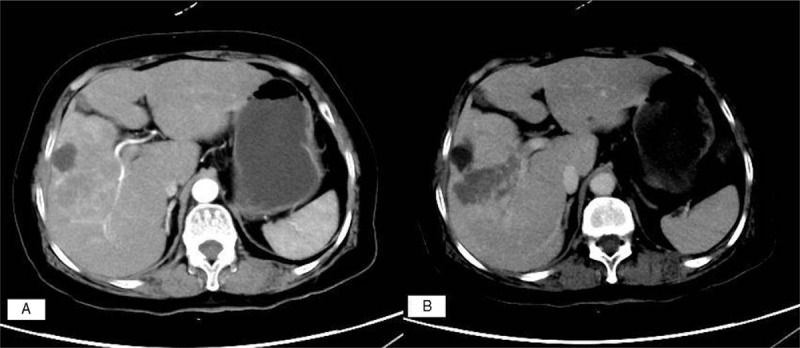
(A) A PHNEN (G3) image in which the tumor-feeding artery is visible. (B) A PHNEN infiltrating a branch of the portal vein.

**Figure 3:**
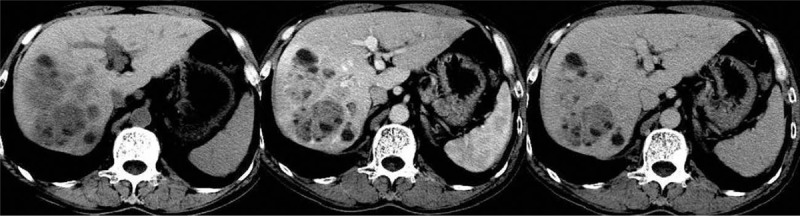
Cystic areas and enhanced solid areas are relatively characteristic imaging finding in PHNEN.

**Figure 4:**
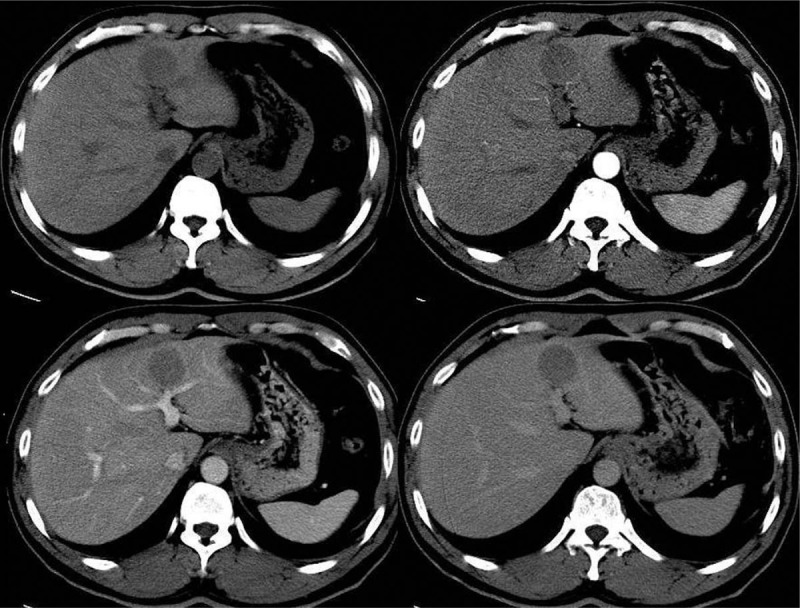
A small round mass in left-lateral lobe, which showed no enhancement in dynamic CT scan.

The preoperative diagnostic techniques of modern hepatology mostly depend on biopsy, imaging, tumor markers, and liver disease background. No evidence show that liver disease background leads to PHNEN. Biopsy was mostly abandoned at the risk of tumor spreading, while malignancy was suspected. Factors, including the similarity to other hepatic tumors in imaging, no specific tumor marker, no related liver disease background, along with the rarity of incidence, make it rather difficult to make a correct preoperative diagnosis for PHNEN.^[20]^ Radiological imaging findings of PHNENs can often be confused with other hepatic tumors. Sometimes, it looks like HCC with contrast agent filled-in in the arterial phase and washed-out in the venous phase (Fig. 1). Or it may be misdiagnosed as iCCA while performing sustained enhancement. Dilated arterial vessels surrounding the tumor could be occasionally observed, which probably fed the tumor (Fig. 2A). Consistent with other primary hepatic malignant tumors, vascular invasion including portal vein or hepatic vein exists in PHNEN (Fig. 2B). PHNEN was occasionally misdiagnosed as cystadenoma due to the central cystic degeneration. Although PHNEN lacks unique performance in dynamic CT/MR scanning, some relatively characteristic imaging features could be learned, including cystic areas with hemorrhagic components and early enhanced solid areas. Those cystic areas appear as nonenhancing small foci on CT and high-intensity foci on T2-weighted MRI (Fig. 3).^[22]^ PHNEN appears to be a hyperechoic mass similar to hemangioma in ultrasound. Histologically, these correspond to small vascular lakes containing fluid and clots that may develop secondary to intratumoral hemorrhage.^[23]^ This phenomenon can be obvious in larger tumors, but not in all smaller ones (Fig. 4). Scintigraphy is an imaging technique providing both diagnostic and therapeutic information in patients with NET. Octreotide scintigraphy (OctreoScan) is used with this aim and has a sensitivity ranging from 85% to 90%.^[24]^ Another benefit of the octreoscan, other than detecting the tumor location, is the ability to predict the response of the tumor to the treatment administered through the octreotide analogues.^[25]^ Unfortunately, octreoscan was not available in our clinic and no patients got this examination.

Serum tumor markers such as CgA and 5-hydroxyindole acetic acid (5-HIAA) are helpful for diagnosis in NEN. As PHNEN seldomly show endocrine function, 5-HIAA probably has a low sensitivity on it.^[26]^ Unlike 5-HIAA test, serum CgA level can be utilized not only in tumors secreting serotonin but also in the diagnosis of atypical or nonsecreting tumors. It was reported that CgA has a sensitivity ranging from 87% to 100%, and a specificity ranging from 84% to 95%.^[27,28]^ CgA is regarded as a particularly useful indicator for assessing prognoses and monitoring disease.

**Figure 5:**
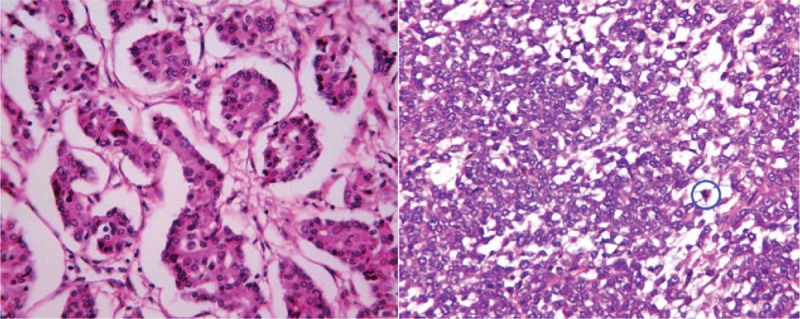
HE×400: (A) Microscopic findings for a PHNEN (G3), revealing tumor cells aligned in a nest-like structure and surrounded by cuboidal cells. Cancer nests were encased by blood sinusoids. (B) Mitosis in the examined circle.

In gross pathologic inspection, the PHNENs appeared as masses of soft tissue with a mix of solid and cystic components and necrotic bleeding. A specific pathological feature with insular, trabecular, or glandular cell arrangements as demonstrated by hematoxylin and eosin (H&E) staining is indicative of an NEN. Immunohistochemical staining was conducted on serial deparaffinized sections. Only Ki67 staining within the nucleus was regarded as a positive result. Additional immunostaining was performed for chromogranin A, neuron-specific enolase, and synaptophysin to differentiate PHNEN from HCC and metastatic carcinoma.^[6,10,29]^ The combination of histomorphological features and immunohistochemical results ultimately support diagnoses of PHNENs (Fig. 5).^[30,31]^ Ki67 labeling index and mitotic grade indicate cellular proliferation are somewhat suggestive of malignant potential. NENs are categorized into 3 groups by grade. Low- and intermediate-grade NENs are classified as grade 1 (G1) and grade 2 (G2) neuroendocrine tumors, respectively, whereas high-grade NENs are known as neuroendocrine carcinomas (G3). We found that for PHNENs, the mitotic rate was often lower than the Ki67 grade for highly proliferative tumors. A total of 10 patients with a Ki67 index greater than 20% had mitotic rates below 20. This phenomenon was observed in 2 out of the 9 G2 patients and all of the G3. When the mitotic rate and Ki67 index indicate different grades, it is suggested that the higher of the 2 grades should be assigned.^[32]^

PHNENs can only be clinically diagnosed after excluding the possibility of NENs of extrahepatic origin with liver metastasis and obtaining definite pathological evidence to support this diagnosis.^[6,21,29]^ So, the diagnosis of PHNET is a continuous process starting from preoperative suspect to postoperative stage, including long-term follow-up to exclude extrahepatic primary origin.^[33]^ Prior reports have indicated that an ultimate diagnosis of PHNEN should not be issued until after 1 year of follow-up.^[34]^ Imaging examination, digestive tract endoscopy, and other examinations to detect potential primary NEN in other organs could be applied. The clinical value of PET/CT in diagnosis and assessment of NEN was formerly thought low due to the low sensitivity of traditional tracer 18F-FDG for well-differentiated NEN.^[35]^ Application of new tracer of 68Ga-somatostatin analogue raised the sensitivity to 82% while singly used and even 92% while combination used with 18F-FDG PET/CT.^[36]^ To date, PET-CT with somatostatin analogs labeled with Ga is increasingly recognized as the best imaging modality for the evaluation of well-differentiated neuroendocrine tumors.^[37]^ Unfortunately, tests for these markers could not be conducted at our hospital. Ultrasonography or CT/MRI is routinely recommended in postoperative follow-up monitoring in most hospitals.

To date, surgical resection remains a mainstay therapy for PHNEN.^[26]^ PHNEN is regarded as a type of slowly growing tumor with low malignancy.^[38,39]^ In our data, 2 patients had found hepatic lesions for more than 2 years and referred here not until the lesions grew. This revealed that PHNEN might have a long natural progress. Cellular proliferation may easily be regarded as a tool accessing tumor malignancy potential in most tumors, but in a particular neoplasm such as PHNEN, it deserves validation. Our analyses indicated that radical resection, less tumor burden (tumor size and TNM stage), and lower cellular proliferation led to longer survival. But only the lower cellular proliferation grade (G1/G2) was demonstrated as a favorable prognostic factor for PHNEN. Zhang et al^[21]^ reported that tumor number was not a prognostic factor for PHNEN, which was partly consistent with ours.

Transcatheter arterial chemoembolization (TACE) and systemic chemotherapy were considered as main therapies for unresectable or recurrent patients, but the outcome was unsatisfactory. Two patients received TACE after recurrence and eventually got the OS after TACE of 6 months for both. Park et al^[17]^ reported that chemotherapy with a combination of fluorouracil, etoposide, and cisplatin produced partial responses in 3 patients; their OS durations were 3.0, 6.2, and 26.4 months. In our study, the OS after recurrence of the 3 patients received mono chemotherapy (fluorouracil or capecitabine-based) were 8, 12, and 28 months, respectively. The evolution of a series of systemic therapeutic options for NEN such as lanreotide and everolimus has emerged over the last few years.^[40–42]^ But to the PHNEN, all information regarding therapeutic regimen were null due to lack of experiences. Another question arouses attention is if a prophylactic appendectomy at the time of hepatectomy was reasonable.^[43]^ Although the primary appendiceal NEN was sometimes insidious, several studies focusing on this disease showed no evidence supporting that it had a high rate of liver metastasis. Thus, a prophylactic appendectomy is not recommended.

Our study exhibited certain limitations. First, this investigation utilized a retrospective study design. The rarity of PHNEN cases prevents us from performing a randomized controlled trial to compare different therapies of choice. Second, most patients in our study have not reached their endpoints; thus, an exact OS could not be calculated for certain patients. Finally, the pathological grading system used in this study was initially designed for NENs from the stomach, small intestine, and pancreas. In principle, PHNENs and NENs from these organs should exhibit similar oncological pleomorphism because the liver is a midgut-derived organ. However, no relevant research has been reported; thus, the question of whether this grading system is suitable for PHNENs merits additional study. Since they were not standard tests in our institute, our study could not provide insights about serous CgA and 5-HIAA, which are important to the preoperative diagnosis and postoperative screening.

## 5 Conclusion

PHNENs are a type of tumor that exhibits slow growth, low-grade malignancy, and relatively consistent imaging results characteristic of mixed solid and cystic hepatic lesions. Diagnoses of PHNENs are dependent on pathological examination, the exclusion of metastasized NENs, and postoperative follow-up. Radical resection of the tumor remains the most effective treatment of choice. It may be reasonable to apply the Ki67 proliferative index to the pathological grading of PHNENs. R0 resection and a G1/G2 histological classification may be favorable prognostic factors for this disease.

## Acknowledgment

The authors are grateful to SHAO Dandan for providing valuable imaging data.

## Author contributions

**Conceptualization:** Changying SHI, Jiamei Yang.

**Data curation:** Changying Shi, Feng Xie.

**Formal analysis:** Changying SHI, Binghua Dai.

**Investigation:** Qian Zhao, Feng Xie.

**Methodology:** Changying SHI, Qian Zhao.

**Project administration:** Jiamei Yang.

**Resources:** Qian Zhao.

**Software:** Binghua Dai, Feng Xie.

**Supervision:** Qian Zhao.

**Validation:** Changying SHI, Binghua Dai.

**Visualization:** Qian Zhao.

**Writing – original draft:** Changying SHI, Binghua Dai.

**Writing – review & editing:** Changying SHI, Feng Xie, Jiamei Yang.

Author name: orcid number
